# Precisely controlled fabrication, manipulation and *in-situ* analysis of Cu based nanoparticles

**DOI:** 10.1038/s41598-018-25472-y

**Published:** 2018-05-08

**Authors:** L. Martínez, K. Lauwaet, G. Santoro, J. M. Sobrado, R. J. Peláez, V. J. Herrero, I. Tanarro, G. J. Ellis, J. Cernicharo, C. Joblin, Y. Huttel, J. A. Martín-Gago

**Affiliations:** 1 Instituto de Ciencia de Materiales de Madrid (ICMM-CSIC), c/Sor Juana Inés de la Cruz, 3, Madrid, 28049 Spain; 2 Centro de Astrobiología (CSIC-INTA), Ctra. de Torrejón a Ajalvir, Km 4, Torrejón de Ardoz, 28850 Spain; 3 Molecular Physics Department, Instituto de Estructura de la Materia (IEM-CSIC), c/Serrano, 123, Madrid, 28006 Spain; 4 Instituto de Ciencia y Tecnología de Polímeros (ICTP-CSIC), c/Juan de la Cierva, 3, Madrid, 28006 Spain; 5 Institut de Recherche en Astrophysique et Planétologie (IRAP), Université de Toulouse (UPS), CNRS, CNES, 9 Avenue du Colonel Roche, 31028, Toulouse, Cedex 4 France

## Abstract

The increasing demand for nanostructured materials is mainly motivated by their key role in a wide variety of technologically relevant fields such as biomedicine, green sustainable energy or catalysis. We have succeeded to scale-up a type of gas aggregation source, called a multiple ion cluster source, for the generation of complex, ultra-pure nanoparticles made of different materials. The high production rates achieved (tens of g/day) for this kind of gas aggregation sources, and the inherent ability to control the structure of the nanoparticles in a controlled environment, make this equipment appealing for industrial purposes, a highly coveted aspect since the introduction of this type of sources. Furthermore, our innovative UHV experimental station also includes in-flight manipulation and processing capabilities by annealing, acceleration, or interaction with background gases along with *in-situ* characterization of the clusters and nanoparticles fabricated. As an example to demonstrate some of the capabilities of this new equipment, herein we present the fabrication of copper nanoparticles and their processing, including the controlled oxidation (from Cu^0^ to CuO through Cu_2_O, and their mixtures) at different stages in the machine.

## Introduction

By reducing the dimensions of a material to the nanometer regime, new physicochemical properties emerge that lead to a wide range of novel applications^1–3^. Currently, a large variety of technological fields, such as catalysis^4–7^, sensing^8,9^ or biomedicine^10,11^ have an increased need for high throughput methods for the production of clusters and nanoparticles (NPs) with a precise control of size, shape and composition. Wet chemistry protocols meet the requirements of high throughput with relative simplicity and low cost. However, they have intrinsic limitations regarding the purity, size distribution and, for particular NP configurations, thermodynamic restrictions also exist. For an extreme control of the reaction conditions and purity of the clusters and NPs produced, gas-phase techniques working in ultra-high vacuum (UHV) present the ideal environment^12^, thanks to the absence of ligands^5^, albeit with lower throughput. Amongst these, sputter gas aggregation sources developed by Haberland and co-workers^13^ have become the most popular, not only due to the fraction of ionized NPs, which allows mass/charge selection^14^, but also to the wide variety of materials that can be sputtered^15^.

In order to overcome the throughput limitation, we have designed and built a scaled-up gas aggregation source. Different approaches have been developed during recent years in order to obtain large NP fluxes with gas aggregation sources^16–19^. In our case, the new design is based on an adjustable multi-magnetron or Multiple Ion Cluster Source (MICS)^20^, which incorporates three independent magnetrons inside an aggregation zone. Although the MICS has emerged as a precise tool for the controlled fabrication of complex NP architectures with adjustable chemical composition, size and structure^21,22^, the approach presented here represents a further step in terms of the tailored generation of controlled nanoparticles with high throughput. The new design of the scaled-up MICS is the origin of the *Stardust* machine, an innovative experimental station devoted to the production of large amounts of NPs, which also offers the possibility of in-flight manipulation (i.e.: gas injection at different stages, heating, accelerating,…) and *in-situ* characterization by electron spectroscopy techniques and thermal desorption spectroscopy.

*Stardust* was originally designed to simulate cosmic-dust formation and processing in the circumstellar region of evolved stars. The ultimate goal is to fabricate in the laboratory the nanoparticles that constitute the refractory part of this cosmic dust. Today with the new generation of telescopes and antennas, such as the ALMA interferometer in Chile, we start to obtain detailed information on the physical and chemical conditions that lead to the formation of this refractory dust^23^. Notwithstanding, the range of use of *Stardust* can be extended far beyond this original conception to many different fields of application in nanotechnology. *Stardust* offers a unique workbench to unravel the properties of individual NPs through in-flight processing and analysis.

In this article, we present a description of the technical solution adopted to build the *Stardust* machine and some representative results to demonstrate its capabilities. In particular, we have focused on the precise fabrication, manipulation and *in-situ* analysis of Cu nanoparticles. One of the most interesting processing capabilities involves the reaction with gases at different stages (or modules) in *Stardust*. In this respect, we have performed a controlled oxidation study of Cu NPs.

Copper-oxide NPs exhibit a different band-gap as a function of stoichiometry and therefore it is possible to tune their optical and electronic properties from metal to semiconductor. Thus, the preparation of high-quality copper-oxide NPs with a narrow size distribution are of increasing interest in fields such as solar energy conversion cells or catalysis^24,25^. Cu based NPs have been previously produced using gas aggregation sources, either for fundamental studies concerning the fabrication process itself^26,27^, or for testing new cluster sources^28^. However, to our knowledge, an accurate control of the Cu oxidation state through reactive sputtering using gas aggregation sources that avoid poisoning of the target has not been previously reported. Here we present a fabrication route based on a sputter gas aggregation source with controlled oxygen injection at different stages of NP formation, using the gas-processing capabilities of *Stardust*, which allows accurate tailoring of the degree of oxidation of the NPs, from metallic Cu through Cu_2_O to CuO. The results are compared with oxidation treatments performed on Cu NPs supported on highly ordered pyrolytic graphite (HOPG).

## Results and Discussion

### The stardust machine

**Figure 1:**
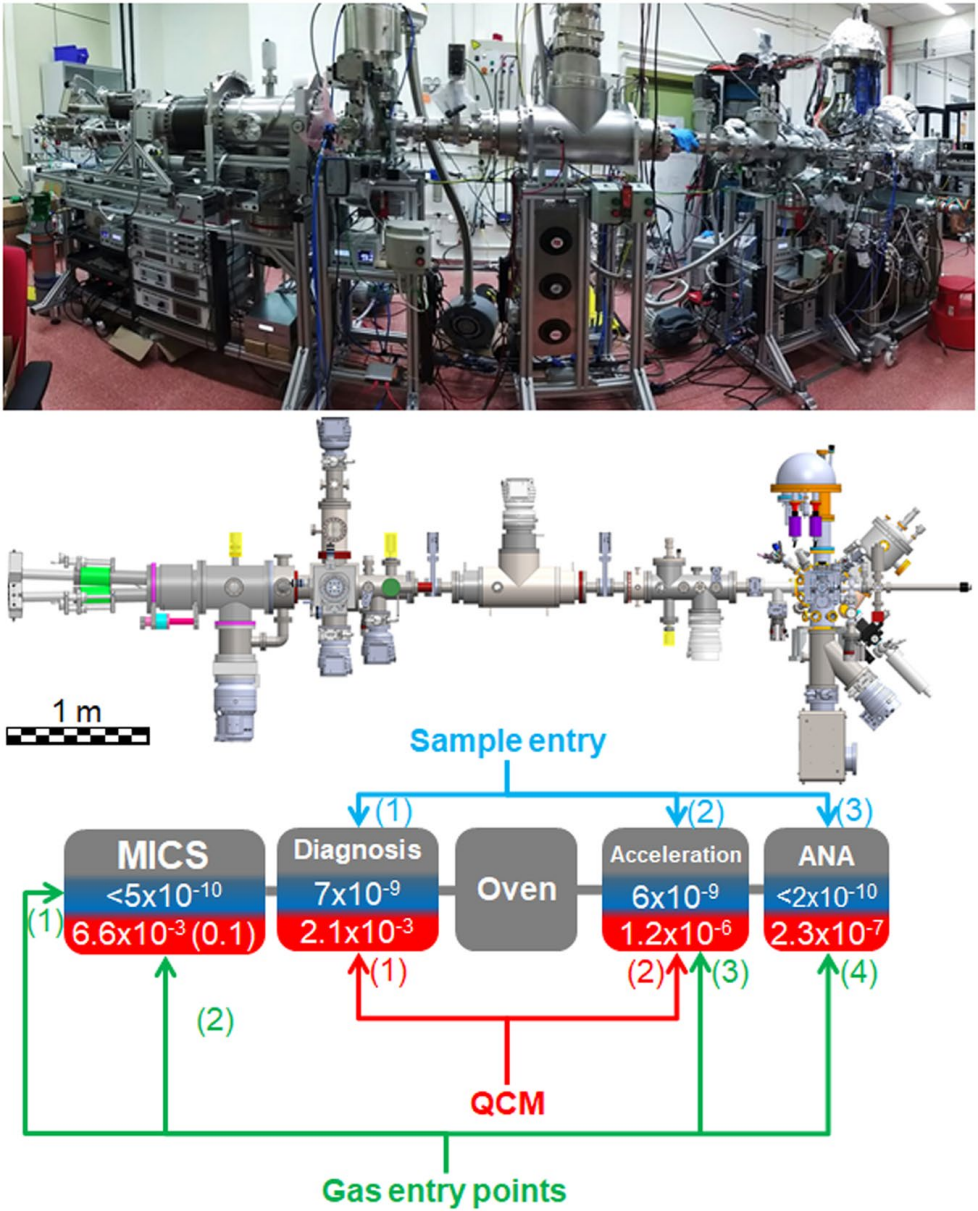
The *Stardust* machine, side view (photograph, top), machine schematic (middle); block diagram and scheme of the pressures, in mbar, in the system (bottom): base pressure on blue background and working pressure on red background (for ϕ_T_ = 150 sccm). The pressures indicated were measured with the gauges highlighted in yellow in the picture. In the case of the MICS module, the value in brackets corresponds to that measured inside the aggregation zone. Gas entry points (1–4) and quartz crystal microbalance position (QCM 1 and 2) are indicated.

In order to provide the maximum flexibility for the fabrication, manipulation and analysis of nanoparticles, *Stardust* was conceived as a modular device. At present it comprises 5 different UHV modules. Figure 1 presents the global design of the equipment in one of its possible configurations.

The first and fundamental module, called the *MICS* module, is a sputtering based gas aggregation source devoted to cluster and NP fabrication. The presence of three completely independent magnetrons inside the aggregation chamber, allows the simultaneous use of up to three different targets for a precise generation of clusters and NPs of different sizes, composition and structure, ranging from single element particles to alloys or core-shell nanostructures, with a precise control of the stoichiometry^21,22,29^. A scaled-up MICS was designed in order to achieve high NP fluxes and larger NP sizes. This new device, fabricated by Oxford Applied Research Ltd. under the name MICS3, employs 3 magnetrons of 2” diameter (instead of 1” from the original design). The aggregation zone where the three magnetrons are located has been re-dimensioned and two facing path-troughs towards the exterior have been included at both sides. These new entrances can be used either for pressure measurements, plasma monitoring or for gas injection. Further information about this module is provided in Section S1 of the Electronic Supplementary Material (ESM).

The NPs produced in the MICS module can be further manipulated, analyzed or collected in other modules. The subsequent three modules are dedicated to the monitoring and modification of physical properties of the generated NPs. They are called *Diagnosis*, *Oven* and *Acceleration* modules.

In the *Diagnosis* module the NPs yield can be monitored using a quartz crystal microbalance (QCM-1 in Fig. 1). It also incorporates a fast entry port for the introduction of substrates, allowing samples to be prepared for *ex-situ* analysis. An electrostatic quadrupole deflector can guide the charged particles towards a quadrupole mass spectrometer. The quadrupole can be placed in line with the NP beam to perform mass filtering. In an initial configuration, it was mounted in line, but to increase throughput we consider the configuration described to be a better option. Full details of this module are given in the ESM, Section S2.

After travelling through the diagnosis chamber the NPs enter the *Oven* module. In their path through this module the NPs are immersed in an IR-bath, absorbing the electromagnetic radiation during their transit, which results in an increase in their temperature. Details of this module are provided in the ESM, Section S3.

The last manipulation module of *Stardust* is the *Acceleration* module, where it is possible to modify the speed and trajectory of the NPs during their travel through it using charged particle optics, and/or induce interactions with injected gases. Just before the exit of this module, a Faraday cup and a quartz crystal microbalance (QCM-2 in Fig. 1) are installed to help monitoring all processing in the chamber. A scheme of this sequence is provided in Fig. S4.1 of the ESM.

Finally, in the fifth module called *ANA*, *in-situ* analysis of the NPs can be undertaken by electron spectroscopy techniques and thermal desorption spectroscopy. A PHOIBOS 100 1D electron/ion analyzer with a one-dimensional delay line detector allows X-Ray photoelectron spectroscopy (XPS), ultraviolet photoelectron spectroscopy (UPS), Auger electron spectroscopy (AES) and low-energy ion spectroscopy (LEIS) to be performed. Thermal desorption spectroscopy (TDS) is performed using a Pfeiffer HiQuad QMG 700 with QMA 400 mass spectrometer, with a mass range of 0 to 512 amu, and a CP 400 ion counter preamplifier (Detection limit 10^−15^ mbar).

With the exception of the MICS module, the disposition of each module in the machine can be modified to adapt the experimental set-up to a particular scientific objective. Thus, they can be placed in the standard configuration, presented in Fig. 1, removed or placed in a different sequence. This design confers high versatility to the whole system. In the near future, an additional module called *INFRA-ICE* will be incorporated. This will be equipped with an infrared spectrometer and a closed-cycle helium cryostat and will further boost the characterization capabilities of *Stardust* allowing both the study of deposited clusters/NPs and ice-analogs using reflection and transmission FTIR spectroscopy.

*Stardust* includes five gas entry points (depicted in Fig. 1), three in the MICS module where the NPs are fabricated, one in the Acceleration module where the NPs are still in the gas phase and one in ANA, where the NPs are collected on a substrate. This broadens the versatility of *Stardust* for NP processing, not only in terms of the modification of the composition of the NPs, but also for heterogeneous catalytic processes. Gas mixing systems have been implemented to allow dosing of gases and liquid vapors (detailed information in Section S5 of the ESM).

In order to have a highly controllable and clean environment for the fabrication of NPs, it is of paramount importance to have a system base pressure that is as low as possible. Special care was taken to use stainless steel gas pipes and CF flanges. The typical base pressures in the system are indicated in Fig. 1, where it is also displayed the typical working pressures for a given sputtering gas injection of 150 sccm. The pumping details are given in the ESM, Section S6. *Stardust* is aligned using a theodolite in order to obtain a fine alignment of all the modules. We have not observed any important deviation of the beam induced by gravity on the particle trajectory.

### Nanoparticle fabrication

#### Rates and size distribution

Cu NPs have been fabricated in order to test the scaling-up of the MICS as well as the processing capabilities of *Stardust*.

**Figure 2:**
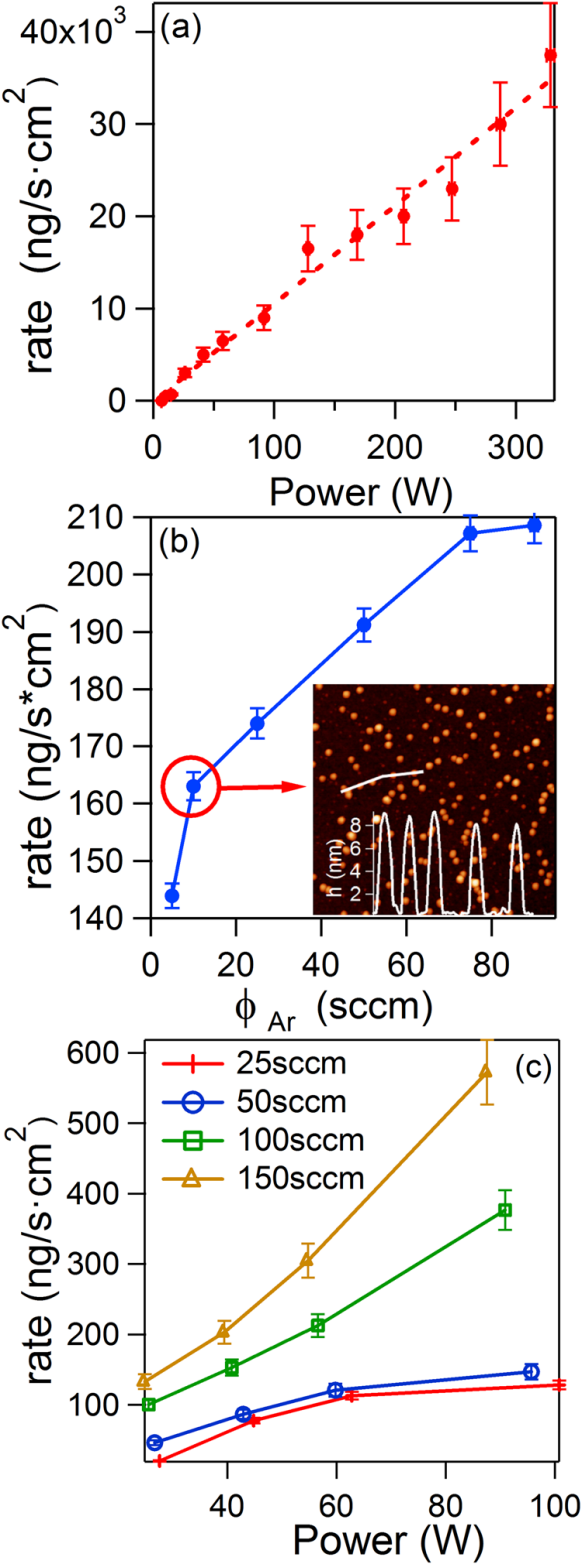
(**a**) Nanoparticle rate vs. power (ϕ_T_ = 100 sccm, ϕ_Ar_ = 50 sccm); (**b**) Evolution of the NP rate as a function of the ϕ_Ar_ injected through the magnetron in use for a ϕ_T_ of 100 sccm (P = 30 W). Inset: AFM image (1 µm^2^) of the sample fabricated with ϕ_Ar_ = 10 sccm. (**c**) NP rate vs. power for different ϕ_T_ in the magnetron in use (ϕ_Ar_ = 10 sccm). The error bars indicate a 10% error of the QCM measurement.

The monitoring of the NP rate generated in the MICS module was undertaken using QCM-1 located at 40 cm from the MICS exit, in the Diagnosis module (see Fig. 1). Figure 2a presents the evolution of the NP rate as a function of the power applied to the magnetron. A continuous increase in the rate with applied power can be observed. Even though these magnetrons can work up to 1000 W, we only tested up to 330 W, reaching a rate of 37.5 µg/s · cm^2^ measured at the beam center, which provides an indication of the high fluxes that can be achieved with this scaled-up MICS.

Besides the well-known operational parameters from conventional sputter gas aggregation sources, such as power, aggregation length (distance of the magnetron to the exit nozzle) or Ar flux (ϕ_T_), the MICS has some characteristic key parameters. For instance, not only is the total amount of sputtering gas injected through the three magnetrons (where ϕ_T_ = ϕ_1_ + ϕ_2_ + ϕ_3_) important, but also the amount of sputtering gas injected through the magnetron in use (ϕ_Ar_ in this work, as only one magnetron is used)^30^. Figures 2b and c display two representative examples. In the first case, the monitoring of the NP rate vs. ϕ_Ar_ evidenced higher rates for increasing ϕ_Ar_. The same trend was found for other ϕ_T_ values. The inset in Fig. 2b presents a representative atomic force microscopy (AFM) image of a deposit fabricated with ϕ_Ar_ = 10 sccm. A narrow size distribution can be observed (8.5 nm, std. dev. 0.3 nm), taking into account that no mass filtering was used.

Figure 2c presents the evolution of the NP rate as a function of the applied power for different ϕ_T_. In this case, for a given power, the higher the ϕ_T_, the larger is the registered rate. It is interesting to notice that for ϕ_T_ = 100 sccm there is a linear increase in the rate with power, similar to that presented in Fig. 2a. However, this linear trend is not followed at other values of ϕ_T_. All the trends presented in Fig. 2 are representative of particular experimental conditions. It must be considered that the evolution of the target during its lifetime, i.e.: the so-called race track formation, can cause variations in the generation of nanoparticles for the same experimental conditions^31^.

#### Optical Emission Spectroscopy in the aggregation zone

**Figure 3:**
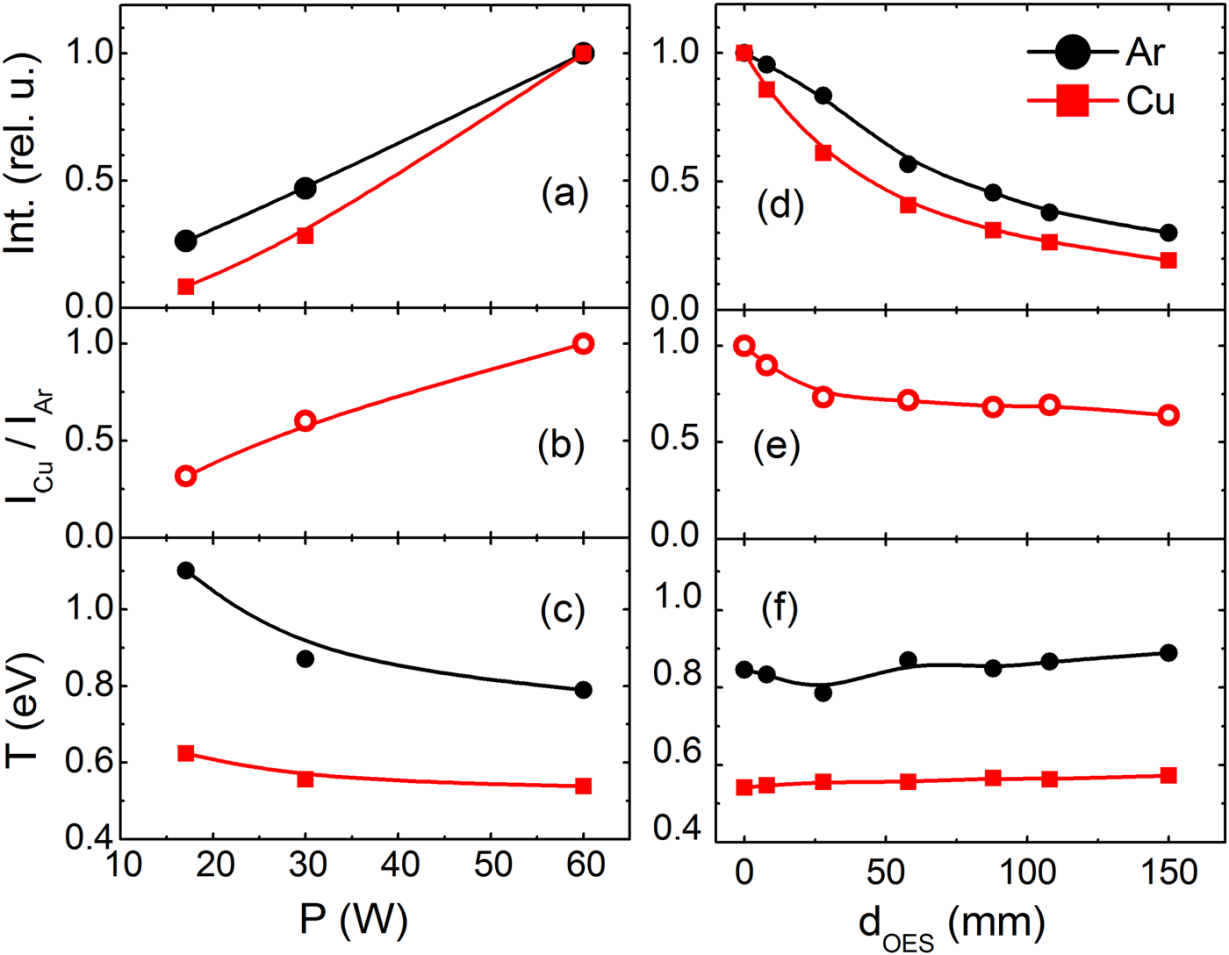
Comparison of the experimental Ar (λ = 696.5 nm) and Cu (λ = 578.2 nm) line intensities (**a,d**), the ratio between both intensities I_Cu_/I_Ar_ (**b,e**) and the Ar and Cu excitation temperatures (**c,f**), depicted as a function of the magnetron power at a fix distance (d_OES = _58 mm) (**a–c**), and as a function of the magnetron distance, d_OES_, at a constant power (P = 30 W) (**d–f**). Lines serve as guides.

By using one of the lateral entrances of the aggregation zone (see ESM, Fig. S1) the plasma can be monitored by Optical Emission Spectroscopy (OES). The optical spectra in the visible range show lines of Ar and Cu neutral atoms. Figure 3a presents the evolution of the intensities of the 696.5 nm Ar line (I_Ar_) and the 578.2 nm Cu line (I_Cu_) as a function of the power applied to the magnetron. For ease of comparison, the signals have been normalized to the maximum power observed. The linear trend observed has also been found in other DC magnetron discharges^32^, and is attributed to the linear increase of electron density with discharge power^33^. The I_Cu_/I_Ar_ ratio also increases with the applied power (Fig. 3b). This quotient is related to the ratio between the excited populations of Cu and Ar^34^ and indicates that the efficiency of Cu sputtering by Ar ions striking the target increases with P. These effects are in accordance with the increase in NP deposition rate with power shown in Fig. 2. Figure 3c illustrates how the excitation temperatures for Ar and Cu, extracted from Boltzmann plots, decrease with power from 1.10 to 0.78 eV for Ar and from 0.62 to 0.50 eV for Cu over the interval investigated. This behavior has been ascribed to the increase of the sputtering yield of Cu by Ar ions, which causes in turn a loss of electron energy in the magnetized region by excitation and ionization collisions with the metal atoms^35,36^.

**Figure 4:**
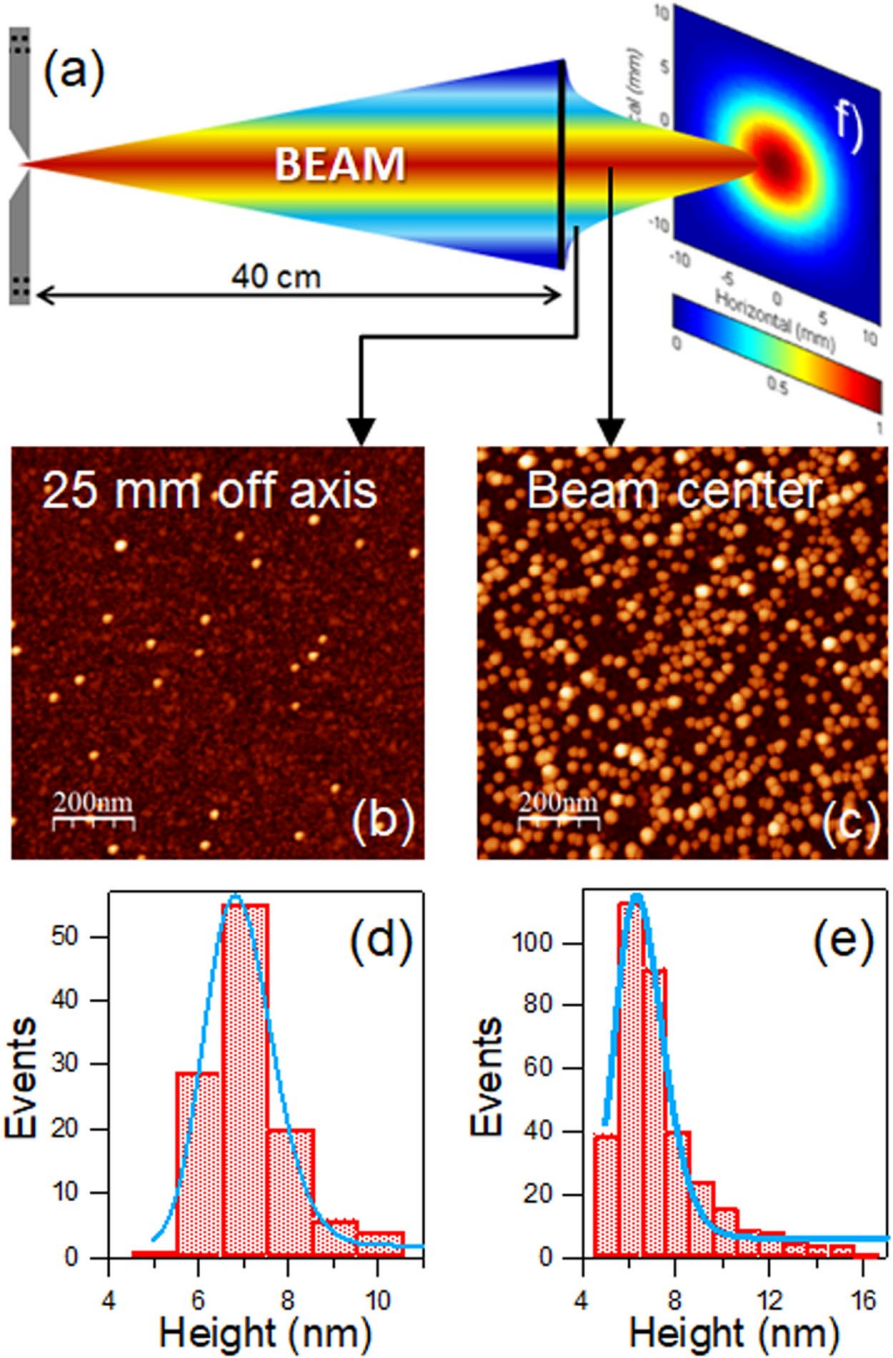
(**a**) Illustration of the beam divergence at the exit of the MICS with the Gaussian profile measured with QCM-1. AFM images of nanoparticles collected (**b**) at 25 mm off axis and (**c**) at the beam center. Log-normal distribution of the height on the nanoparticles collected (**d**) at 25 mm off axis, and (**e**) at the beam center. (**f**) Beam shape derived from the optical absorption measured in the mid-IR region of nanoparticles deposited on a glass slide at 740 mm from the MICS exit.

Figure 3d presents the evolution of I_Ar_ and I_Cu_ as a function of the distance between the magnetron and the line of sight of the optical spectrometer (d_OES_). Both intensities decrease with increasing d_OES_ due, most likely, to the fall in electron density further away from the magnetron^37^. The evolution of the ratio of the normalized intensities I_Cu_/I_Ar_ is presented in Fig. 3e. Over the first 30 mm this ratio drops by ∼30% suggesting an initial dilution of the sputtered Cu atoms with increasing distance from the Cu target. Aggregation processes could also contribute to the observed drop. Beyond this point, the I_Cu_/I_Ar_ ratio shows only a slow decline. The excitation temperatures of Ar and Cu as a function of d_OES_ are analyzed in Fig. 4f. In both cases the temperature is approximately constant (variations < 10%) over the range of distances studied. This observation indicates that the electron energy distribution, which essentially determines the population of the excited states leading to the observed emissions, should not vary appreciably over the same distance interval. In fact, the electron temperatures measured by Langmuir probes in conventional magnetron plasmas^33,38^, have been found to remain constant for a relatively large distance from the magnetron.

#### Beam characterization

The beam shape after the expansion out of the MICS module was evaluated with the QCM-1 mounted on a z-translator perpendicular to the NP beam. The results showed a Gaussian beam shape with a divergence of around ±5° under the experimental conditions tested (Fig. 4a). The homogeneity of the NPs produced with the MICS was analyzed both at a position 25 mm off-axis (Fig. 4b) and at the beam center (Fig. 4c) by AFM, on samples deposited through the sample entry-1 (located in front of QCM-1). It can be observed that for the same deposition time of 2 s the rate clearly decreases off-axis. The mean nanoparticle size follows a typical log-normal distribution, with the maximum at 6.8 nm when measured off-axis (Fig. 4d) and at 6.3 nm in the beam center (Fig. 4e). The presence of an increased number of larger NPs (tail of the log-normal) when measured at the beam center probably arises from the fact that, as can be observed in the AFM images, the NP density is much higher in this sample and some particles could be found on top of others. Moreover, in the AFM images the presence of small features can be observed, which might be confused with small nanoparticles. However, an AFM analysis of the as-received SiOx substrate shows that they are induced by the surface roughness as these features are present prior to deposition of NPs. Additional information on this issue is provided in Section 7 of the ESM.

For an improved characterization of the beam shape, we deposited NPs on glass slides and performed subsequent *ex-situ* measurements of the optical absorption in the mid-IR region. Figure 4f (right-hand side of Fig. 4a) presents the resulting homogeneous 2D-gaussian profile.

Manipulation of the NP beam is possible using the acceleration module (see ESM, Fig. S4.1). By placing QCM-2 in the beam axis and the faraday-cup 20 mm off-axis, when a voltage is applied to the second set of deflection plates, a decrease in the NP yield of about 60–80% was registered in QCM-2. This means that for the experimental conditions employed (P = 30 W; ϕ_T_ = 150 sccm; ϕ_Ar_ = 10 sccm; aggregation length 374 mm), around 60–80% of the mass of the Cu NPs produced in the MICS module is carried by charged particles. From the sign of the current detected with the Faraday cup, one can determine that they are mostly negatively charged. Moreover, as the ionizer at the entrance of the acceleration module can be operated to further ionize neutral species, it is possible to further increase this percentage (see ESM, Fig. S4.2). The use of the grid located at the beginning of this module increase in the NP yield of between 2.5 and 3.5 times measured at QCM-2. Finally, by applying −180 V to the Einzel-lens after the grid, an increase of more than 50% in the NP yield was also observed. It is worth mentioning that, although the fraction of Cu charged NPs measured could be as high as 80%, only a very small number of charged atoms are involved. Indeed, considering that the NPs are singly charged and the average diameter of the NPs is 7 nm in these experiments, the ratio of charged to neutral atoms is of the order of 10^−5 ^^20^.

The average speed of the charged Cu NPs was determined by applying a trigger voltage on the first set of deflection plates at the entrance of the acceleration module and measuring the time delay on the Faraday cup. An average speed of 140 ± 20 m/s was found for Cu NPs (see ESM, Fig. S4.3). This value corresponds to kinetic energies of the order of a few meV/atom, well within the range for soft landing, ensuring no collision-induced deformation upon collection on a substrate, as expected when using sputter gas aggregation sources^39^.

### Controlling The Stoichiometry Of Cu-Oxide Nanoparticles

The high fluxes of NPs generated with the MICS module make possible *in-situ* characterization in the ANA module, at 4.5 m from the MICS exit. In order to evaluate the effect of gas injection (O_2_ in this case) using the gas mixing system (see ESM, Section S5) at different parts of the *Stardust* machine, Cu NPs deposits were obtained on HOPG substrates located in the ANA module for further XPS analysis.

Three different experiments were performed: firstly, oxidation of Cu NPs supported on HOPG in the ANA module (through gas entry-4); secondly, oxidation through the new entrances performed in the aggregation zone (gas entry-2); thirdly, oxidation of the Cu NPs from behind the magnetrons (gas entry-1).

A power of 40 W was applied to the Cu magnetron without O_2_ injection and was typically controlled in current (0.15 A). The O_2_ injection in the aggregation zone led to an increase in the discharge voltage, an effect that has been previously reported for reactive sputtering in gas aggregation sources^40^.

**Figure 5:**
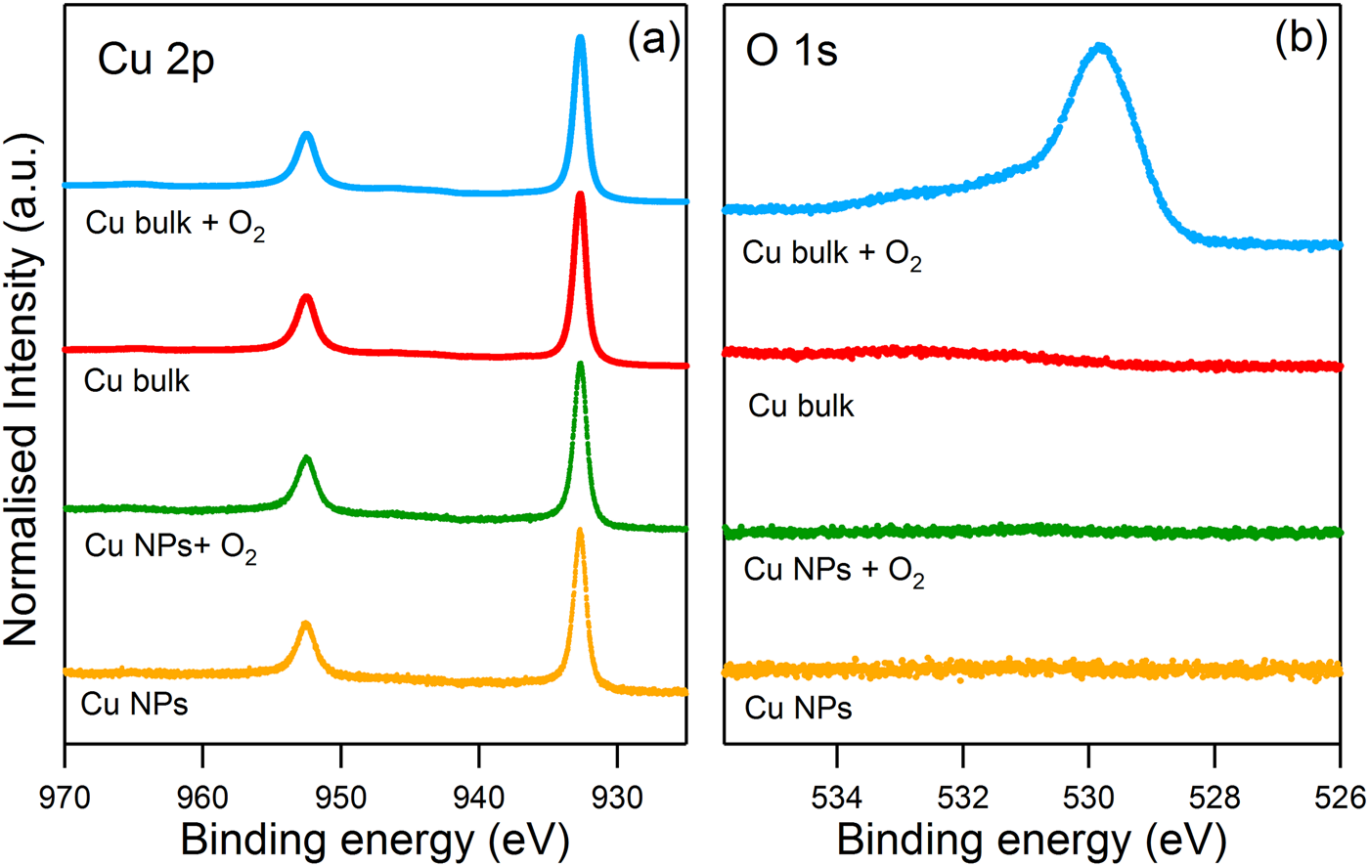
(**a**) Cu 2p and (**b**) O 1 s core level XPS spectra of Cu NPs as-deposited (yellow), after O_2_ treatment in ANA module (green), UHV cleaned Cu bulk (red) and Cu bulk after the same O_2_ treatment (blue).

For the first experiment, Cu NPs were deposited on HOPG in the ANA chamber. Figure 5 shows (a) the Cu 2p and (b) the O 1 s core level peaks for this sample (yellow line). The Cu 2p_3/2_ peak at a binding energy (BE) of 932.7 eV, typical of metallic Cu^41^ and the absence of a peak in the O 1 s core level peak, is a clear indication of the cleanliness of the fabrication process in *Stardust*. The base pressure below 5 × 10^−10^ mbar is important to avoid any possible oxidation from impurities^42^. After O_2_ exposure (13500 L) of these Cu NPs (green spectra in Fig. 5), no evidence of changes in either the Cu 2p or in the O 1 s line shape with respect to the as-deposited NPs was observed, indicating that oxygen does not bind to or react with the NPs. No traces of oxygen were found, even when the O_2_ treatment was performed at 300 °C.

For comparative purposes, a reference Cu sample (hereafter denominated *Cu bulk*) was also measured under the same conditions. After cleaning the sample, the XPS spectra presented an intense Cu 2p peak and the clear absence of oxygen contamination (red spectrum in Fig. 5). By performing the same O_2_ injection in the ANA chamber (13500 L) (blue spectrum in Fig. 5) the emergence of a peak in the O 1 s core level was clearly observed, with a maximum at 529.8 eV and without apparent changes in the Cu 2p peak. Both facts reveal the formation of Cu_2_O^43^. This evidenced the higher chemical inertness of Cu NPs (NP mean size: 7 nm diameter) in comparison to Cu bulk, where a high oxygen dose does not succeed in forming any oxide or adsorbed species.

After the experimental confirmation of the lack of oxidation in the case of Cu NPs compared to Cu bulk in these experimental conditions, a second oxidation experiment was carried out introducing O_2_ in the aggregation zone through gas entry-2. The distance between the magnetron and the gas entry-2 was 180 mm for these experiments. Different ϕ_O2_ fluxes ranging from 0.1 to 21.5 sccm with respect to a ϕ_T_ = 150 sccm were tested in order to evaluate, firstly, the possibility to oxidize the NPs from this new entrance and, secondly, the ability to control the stoichiometry of the oxides generated.

**Figure 6:**
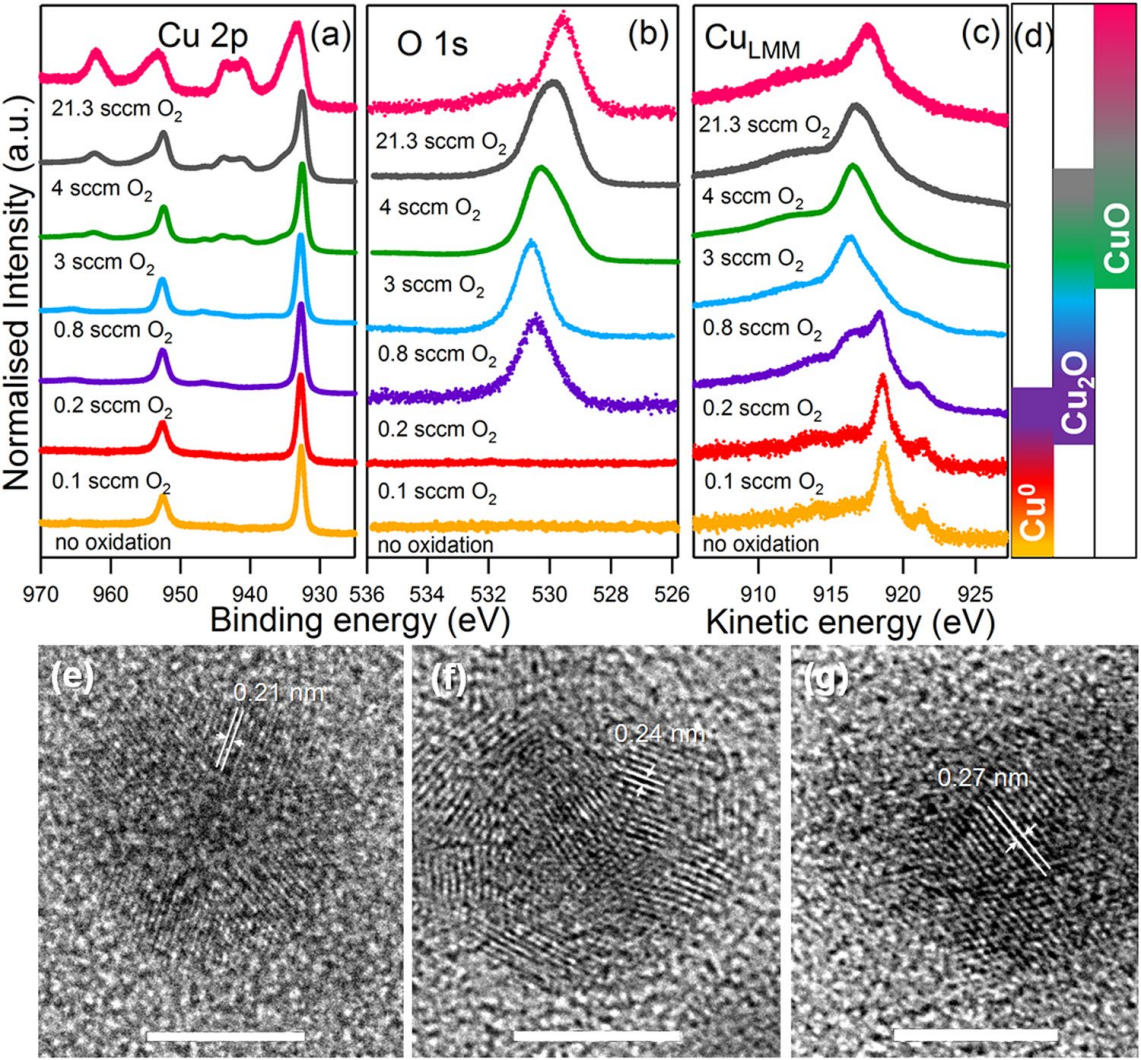
(**a**) Cu 2p, (**b**) O 1 s core levels and (**c**) Cu_LMM_ Auger peak of Cu NPs after different O_2_ treatments using gas entry point −2. (**d**) Diagram of the formation and coexistence of the Cu species with ϕ_O2_. TEM images of (**e**) Cu^0^, (**f**) Cu_2_O and (**g**) CuO nanoparticles. Scale bar: 5 nm.

Figure 6(a–c) displays the line-shape of the Cu 2p (a), O 1 s (b) and Cu_LMM_ (c) of Cu NPs fabricated with different ϕ_O2_ in the aggregation zone. The reference Cu^0^ NPs discussed in the previous figure (yellow curve) is also included in Fig. 6 for ease of comparison. The injection of 0.1 sccm O_2_ in the aggregation zone (red curve) does not seem to induce any detectable change in the Cu NPs in comparison to Cu^0^ NPs. However, higher values of ϕ_O2_ have a progressive influence on the final composition of the NPs. The value of 0.2 sccm O_2_ (violet curve) constitutes the threshold ϕ_O2_ to initiate oxidization of the Cu NPs fabricated under the experimental conditions employed. Even though the Cu 2p core level has the same shape as Cu^0^ NPs, as previously discussed in Fig. 5, the presence of a peak in the O 1 s core level evidenced the formation of Cu_2_O. The same was observed for a flux of 0.8 sccm (blue curve), where only a slight shift of 0.1 eV towards higher BE was registered in the O 1 s peak in comparison to the 0.2 sccm treatment. The analysis of the Cu_LMM_ peak (Fig. 6c) showed how, whilst the treatment at 0.1 sccm O_2_ presents the same Auger shape as the Cu^0^ NPs, higher O_2_ fluxes induce a change in the Cu_LMM_ peak. For a flux of 0.8 sccm, the shape of the Auger peak corresponds to Cu_2_O^44^, whereas the treatment at 0.2 sccm represents an intermediate situation between Cu^0^ and Cu_2_O, suggesting a partial oxidation of the NPs.

For increasing values of ϕ_O2_, there is a progressive change in the shape of the Cu 2p core level, with a shoulder in the main peak at 934.5 eV and new peaks at 941.3 eV and 943.9 eV. These observations are characteristic of CuO^43,44^ that, for ϕ_O2_ of around 3–4 sccm coexists with Cu_2_O. There is also evidence for CuO formation in the O 1 s core level, with the presence of a component at 529.7 eV and a shoulder at higher KE in the Cu_LMM_ peak. Total Cu oxidation to CuO is achieved using a ϕ_O2_ of 21.3 sccm.

This ability to control the oxidation state of the NPs is confirmed by the structural analysis obtained by high resolution transmission electron microscopy (HR TEM) images. Figure 6e–g shows NPs fabricated (e) in absence of O_2_, (f) 0.5 sccm O_2_ and (g) 21.3 sccm O_2_. The respective interplanar distances indicated in the figure are 0.21, 0.24 and 0.27 nm in accordance to the lattice spacing of (111) Cu^45^, (111) Cu_2_O^46^ and (110) CuO^47^, respectively. These images show the crystalline nature of both, metallic and oxide NPs. Interestingly, in the case of the Cu^0^ image (Fig. 6e), we do not find any appreciable oxidation of the nanoparticle surface induced by molecular oxygen after the *ex-situ* analysis, in good agreement with the XPS results after the oxidation treatment with molecular oxygen in the analysis chamber (Fig. 5).

**Figure 7:**
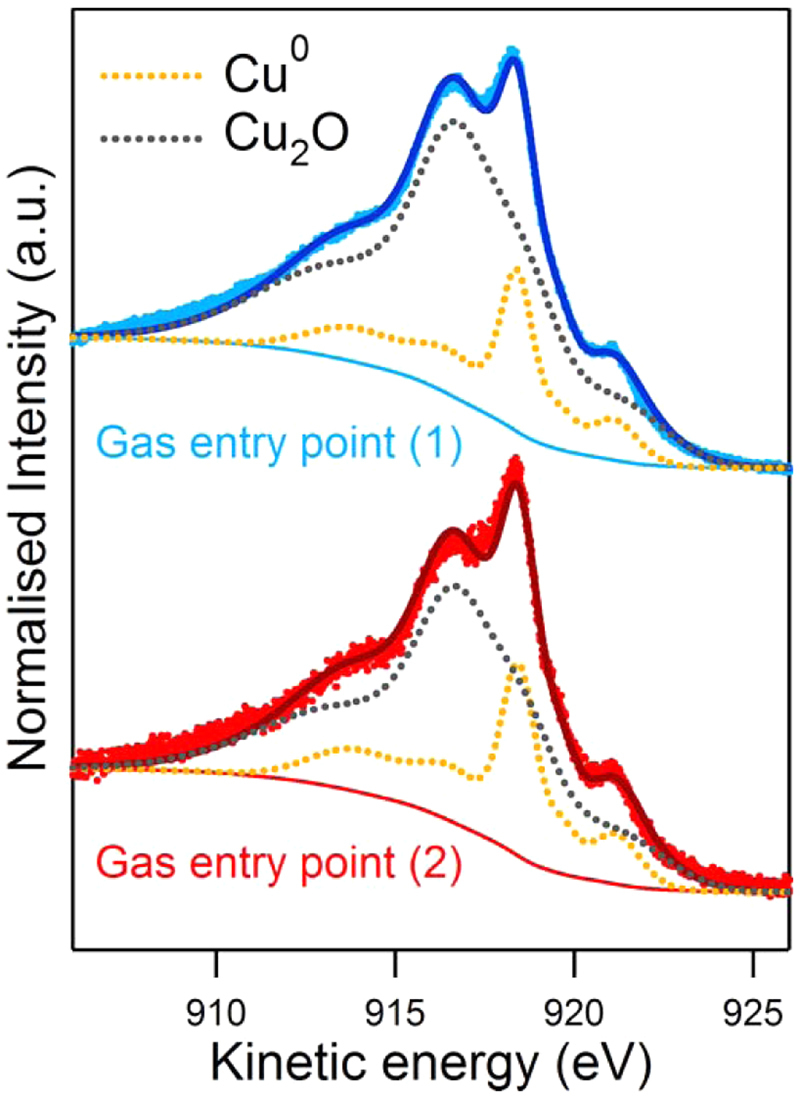
Comparison of the Cu_LMM_ peak after O_2_ injection through gas entry points 1 and 2.

To evaluate whether any difference exists between injection of the reactive gas through gas entries -1 or -2, we injected the threshold ϕ of 0.2 sccm through gas entry-1 (behind the magnetron). This resulted in no significant differences in the Cu 2p and O 1 s core level peaks. Only small changes were observed in the Cu_LMM_ peak (Fig. 7) which indicated a more effective oxidation when introducing the O_2_ from the rear side of the magnetron (77% Cu_2_O in comparison to 70% Cu_2_O through gas entry-2).

It is well-known that oxygen inclusion during fabrication of metal NPs with gas aggregation sources strongly influences the formation process. For metals like Cu, Ti, Al or W, the presence of small amounts of oxygen lead to an increase of the deposition rate^40,48–50^. However, in those cases there is a critical threshold above which this effect vanished due to poisoning of the target, leading to a drastic reduction in the production rate. The results presented here are in contrast with the existing literature since, for all ϕ_O2_ tested, the rates monitored with the QCM are much higher than those in the absence of O_2_ (no values are shown as the rates registered were too high to properly be measured them without causing a crystal failure of the QCM). The differences observed may arise from the manner in which the O_2_ is injected. Reactive sputtering in gas aggregation sources is usually carried out by using a mixture of O_2_ and Ar as sputtering gas. In our case, the entrance for the sputtering gas is left only for Ar, whilst O_2_ is injected in all cases at a distance that seems to be far enough away to avoid target poisoning. However, it is important to point out that the discharge voltages always increased after O_2_ injection, which is indicative of oxygen involvement in the plasma.

In order to evaluate to what extent oxygen can be involved in the sputtering process, OES was employed and spectra were recorded during the fabrication of NPs with ϕ_O2_ = 0 sccm and ϕ_O2_ = 21.3 sccm through gas entry-2. For these measurements, we placed the magnetron closer to the line-of sight of the optical spectrometer (the same as gas entry-2, d_OES_ = 0 mm) to improve the signal-to-noise ratio. This configuration could slightly overestimate the influence of O_2_ with respect to the oxidation experiments discussed in Fig. 6.

**Figure 8:**
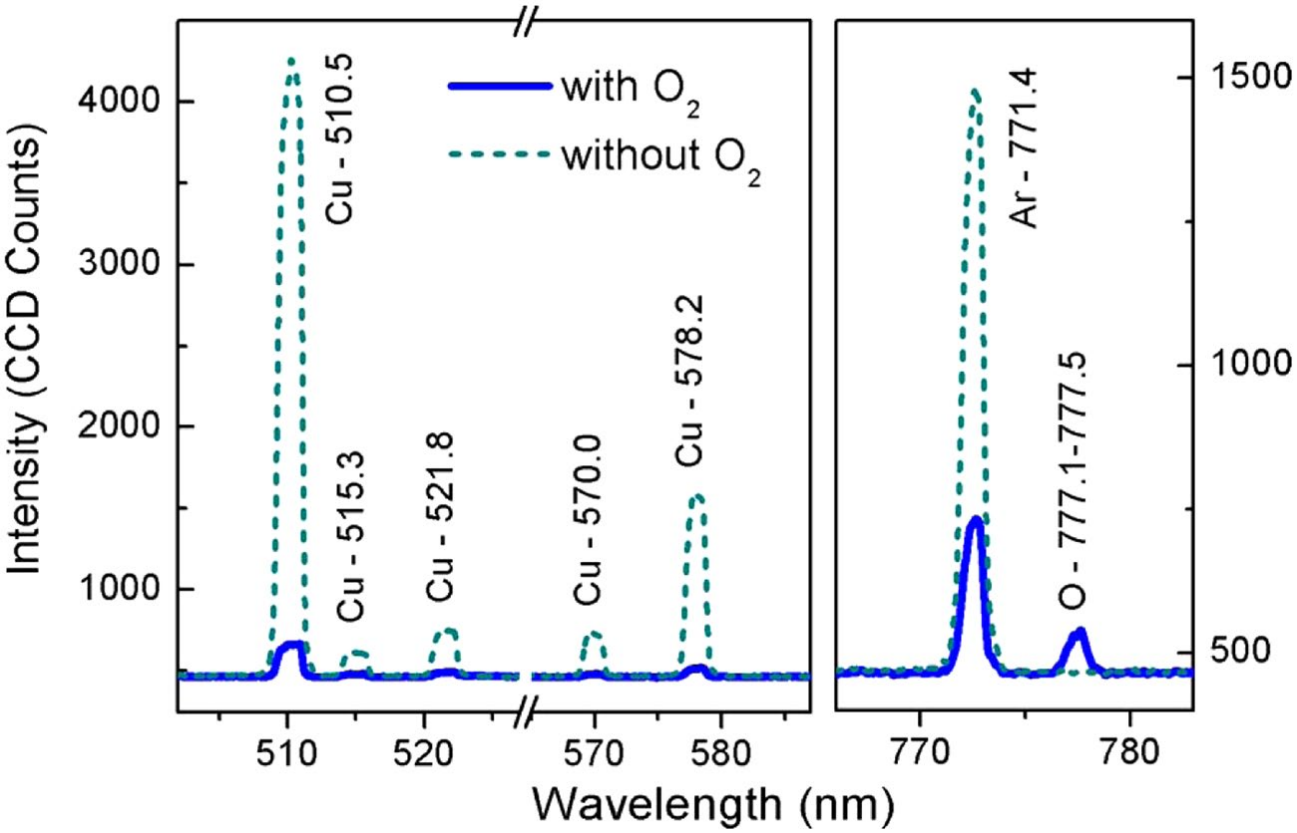
Emission spectra of the magnetron plasma showing the intensity decrease of Cu and Ar lines, and the appearance of excited atomic oxygen, when O_2_ was added through gas entry-2.

The comparison of both spectra, with and without O_2_ (Fig. 8), revealed the appearance of some lines characteristic of atomic oxygen around 777 nm when O_2_ was introduced in the aggregation zone, meaning that dissociation of O_2_ occurs during the process. At the same time, the lines of atomic Cu almost disappeared and the intensity of Ar lines decreased significantly. Similar trends have been previously observed for metal oxide nanoparticles formation using this technique^50^. However, in these cases, the detection of O atoms was correlated to a drastic decrease in the deposition rate, a phenomenon that was not observed here. The formation of Cu oxides, as detected in the XPS analysis, is favored by the presence of free O atoms, which at the same time explains the significant decrease in the Cu emission. Additionally, the consumption of part of the power applied to the magnetron in the dissociation of O_2_ can also contribute to the reduction of Cu emission and the decrease of Ar intensity.

### Summary

In this work, we present the Stardust machine, an innovative UHV experimental station for the production of controlled NPs, with in-flight manipulation and processing capabilities along with *in-situ* analysis of the NPs. The new design of the scaled-up MICS has successfully reached the goal of a high-throughput fabrication of NPs using a sputter gas aggregation source. From the results presented here, deposition rates of 114.5 µg/s·cm^2^ integrated over the Gaussian beam shape were obtained in the case of Cu NPs using only one magnetron operated under conservative fabrication conditions, which correspond to around 10 g/day. Considering that the power applied can be three times higher than the highest power tested in this work, and higher efficiencies can be achieved using other combinations of ϕ_T_ and ϕ_Ar_, this new design represent a significant step forward towards industrial implementation of gas aggregation sources^15,16,51^, for applications with special requirements such as controlled purity, size distribution, stoichiometry and structure. These nanoparticles could be dispersed when deposited on low-interacting surfaces, as graphite. Different approaches have been carried out during the last years in order to disperse these high-quality nanoparticles in liquid media for applications as biomedicine^10,11^. Some of them involve the co-deposition of water vapor for the formation of ice^52^ or in-flight plasma polymer coating^53^.

A homogeneous 2D Gaussian shape beam with a narrow size distribution of the NPs is achieved without any filtering of the NPs (mass/charge selection). The ratio ions/neutrals typically range from 0.6 to 0.8, the Cu NPs being negatively charged. Taking advantage of some of the capabilities for *in-situ* manipulation, we have proven that the use of the ionizer on the Acceleration module increases this percentage of charged NPs. Furthermore, in these conditions the use of the grid and the Einzel lens increase the NP rate more than a 50%, thanks to the acceleration and focusing effect. These tools would lead as well to an increase in the NP rates discussed above.

Gas injection at different stages of Stardust evidenced differences depending on the gas entry point used. This versatility opens the route to reactions with gases at different stages of the formation of NPs, during their flight along Stardust or supported on substrates. Here, using the oxidation of Cu NPs as a showcase, an exquisite control of the stoichiometry of the resulting NPs was achieved when the oxygen is injected in the aggregation chamber (during NP formation). On the contrary, once the Cu NPs are supported on HOPG, it is not possible to oxidize the Cu NPs in the experimental conditions tested.

Finnaly, with the design and construction of Stardust, we have assembled several techniques that combine the fine control of NP fabrication with high throughputs, in-flight manipulation and *in-situ* characterization. The result is a unique workbench not only for creating analogues of stardust particles in the laboratory, but also for basic research including *in-situ* studies of fundamental properties of supported and non-supported NPs. At the same time, the high throughputs already achieved with this new MICS design open up the use of this machine for applied studies, such as catalysis, and further applications in nanotechnology.

## Methods

### Nanoparticle fabrication

Nanoparticles are fabricated with a scaled-up MICS working in UHV (see ESM, Section S1). For the experiments presented in this work, only one of the three magnetrons was used. This magnetron was loaded with a copper target of 99.99% purity, 2” diameter and 4 mm thickness. The results presented in this work were obtained with a nozzle of 9 mm and a diaphragm of 12 mm.

Different total Ar fluxes (ϕ_T_) were used for the experiments, ranging from 25 to 150 sccm. ϕ_T_ corresponds to the sum of the Ar injected through each of the three magnetrons (ϕ_1_, ϕ_2_ and ϕ_3_), even if only one of them is in use. Typical working power was 30 W and the typical aggregation length (distance between the magnetron and the exit nozzle) was 374 mm. The substrates employed for collecting the NPs are boron-doped Si(100) with its native oxide for AFM analysis and HOPG for XPS analysis.

For the gas entry tests at different stages in the *Stardust* machine, extra-pure O_2_ (99.999%) was used. The gas is injected using the gas mixing system described in Section S5 of the ESM. Three different experiments were performed. One consisted in the oxidation of Cu NPs supported on HOPG at a pressure of 1 × 10^−5^ mbar O_2_ for 30 min, which represents about 13500 Langmuir (1Langmuir, L = 10^−6^ mbar·sec). This experiment was carried out in ANA module with the gate valve, which connects this module to the rest of *Stardust*, closed. The other oxidation treatments were carried out during the NPs during formation. In these cases the oxygen flux ϕ_O2_ varied from 0 to 21.3 sccm. For all oxidation tests, the Ar flux was ϕ_T_ = 10 + 70 + 70 = 150 sccm, with 10 sccm corresponding to the Ar injected through the magnetron in use (ϕ_Ar_). The aggregation length was 374 mm and the deposition time 1 min.

### Nanoparticle characterization

#### Optical emission spectroscopy (OES)

The light emitted by the plasma of the Cu target of the magnetron placed closer to the window through which the OES measurements were taken (experimental conditions: P = 30 W, ϕ_T_ = 70 sccm, ϕ_Ar_ = 20 sccm) was analyzed by optical emission spectroscopy (OES) using a grating spectrometer (Ocean Optics, model QE65000) with a 300 grooves/mm grating, a cooled linear CCD-array detector (Hamamatsu S7031-1006) and an optical fiber (QP600-2-SR-BX) coupled to the 25 μm input slit of the spectrometer. The spectral range of the instrument is 200–980 nm, with 0.8 nm spectral resolution. The relative spectral sensitivity, with which the measured intensities were corrected, was calibrated using a reference standard lamp. Plasma emission was collected through an optical window installed in one of the additional entrances to the aggregation zone of the MICS chamber. The axis of the optical system was perpendicular to the discharge axis, and the diameter of the light observation cone in the discharge region did not exceed 1 cm. To estimate the excitation temperatures by means of Boltzmann plots, Ar emission lines were studied in the 675–978 nm spectral interval, which correspond to emissions from 4p, 4d and 5s upper levels, with energies between 12.9 eV and 15 eV; Cu lines were studied in the 465–580 nm range, corresponding to transitions from 4p, 4d and 5s upper levels, with energies between 3.8 eV and 7.7 eV. Energy levels and transition probabilities were retrieved from the NIST Database^54^.

The measurements during O_2_ injection were performed for the experimental conditions of: ϕ_T_ = 150 sccm; ϕ_Ar_ = 10 sccm; ϕ_O2_ = 21.3 sccm and the magnetron was placed in the line of sight of the optical spectrometer and the O_2_ injection took place through the same orifice.

#### X-ray Photoelectron Spectroscopy

XPS measurements were carried out *in-situ* in the Analysis chamber using a PHOIBOS 100 1D electron/ion analyzer with a 1-dimensional delay line detector and a monochromatic Al Kα anode (1486.6 eV). The NPs were deposited for this analysis on freshly cleaved HOPG annealed overnight at 550 °C. The Cu 2p, O 1s core levels and Cu_LMM_ were recorded with a pass energy of 15 eV and a step of 10 meV. The binding energy (BE) scale was calibrated with respect to the C 1s core level peak at 284.5 eV in the case of Cu NPs and at 932.7 eV in the case of the Cu bulk reference sample.

Cleaning of Cu bulk (used as reference sample) was performed by hot sputtering at 580 °C for 30 min using a p_Ar_ = 5.0 × 10^−6^ mbar, 1.5 kV and 12.4 µA.

#### Atomic Force Microscopy

Nanoparticles were deposited on Si(100) wafers in order to perform *ex-situ* characterization by Atomic Force Microscopy (AFM). These substrates present a very low surface roughness, thus allowing a correct height characterization of the deposited NPs. The height and diameter of the NPs are assumed to be equal since the NPs soft-land on the substrates^6^. AFM measurements were performed using the Cervantes AFM System equipped with the *Dulcinea* electronics from Nanotec Electronica S.L. All images were recorded and analyzed using WSxM software^55^.

#### Infrared absorption

*Ex-situ* measurements of the optical absorption in the mid-IR region were carried out by depositing Cu NPs on glass slides. The measurements were carried out using a globar as IR source and focusing the IR beam down to (1.9 × 1.6) mm^2^ (Horiz. × Vert.). The voltage on a liquid nitrogen cooled MCT detector was monitored while scanning the sample using the oversampling technique.

#### Transmission electron microscopy

Transmission electron microscopy (TEM) micrographs were recorded using a TEM/STEM (JEOL 2100F) microscope operating at 200 kV. Amorphous carbon-coated TEM grids were employed for deposition of NPs and subsequent TEM analysis and the images recorded *ex-situ*.

## Electronic supplementary material

Supplementary information
