# Supplementary material for: Precisely controlled fabrication, manipulation and in-situ analysis of Cu based nanoparticles
Source: Sci Rep. 2018 May 8;8:7250. doi: 10.1038/s41598-018-25472-y (PMC5940906; doi:10.1038/s41598-018-25472-y)
Supplement: Supplementary file 1 — Supplementary information [file 41598_2018_25472_MOESM1_ESM.pdf]

Precisely controlled fabrication, manipulation and *in-situ* analysis of Cu based nanoparticles

L. Martínez[1], K. Lauwaet[1], G. Santoro[1], J. M. Sobrado[2], R. J. Peláez[3], V. J. Herrero[3], I. Tanarro[3], G. J. Ellis[4], J. Cernicharo[1], C. Joblin[5], Y. Huttel[1], J. A. Martín-Gago[1*]

ELECTRONIC SUPPLEMENTARY MATERIAL

S1. THE MICS MODULE

This scaled-up MICS employs 3 magnetrons of 2” diameter (instead of 1” from the original design) assembled in a DN 250 CF flange with a gas inlet in the center that is mainly dedicated to He injection. They are direct current (DC) and radiofrequency (RF) compatible in such a way that combinations of conductive and isolating materials can be sputtered. Each of the three magnetrons has three gas entrances, as the standard NC200-U nanocluster source: one for the sputtering gas that injects the gas closely confined parallel to the target surface, and two for injection of other gases during sputtering, with exits behind each magnetron. These additional gas entry points can be used to modify the composition of the NPs during their formation process and are referred to as gas entry - 1 in Figure 1.

The aggregation zone where the three magnetrons are located has been re-dimensioned to house larger magnetrons (13¼" flange, 413 mm length). The lateral pathways performed in the aggregation zone which are connected to outer DN 63 CF windows through 12 mm inner-diameter tubes (see inset, Fig. S1). On the one hand, these new entrances allow a direct measurement of the pressure inside the aggregation chamber, apart from the measurement in the double-wall region of the differential pumping. Typical working pressures in this zone are of the order of a few tenths of mbar. On the other hand, the new entrances can be used for plasma monitoring (see inset of Fig. S1). Currently the atomistic mechanisms operating in the aggregation chamber, responsible for NP growth, are not fully understood and, often, only calculations on postulated processes are undertaken. Plasma monitoring during deposition is possible using a Langmuir probe [1] or optical emission spectroscopy [2] in the aggregation zone. In our set-up, both options are available. Furthermore, these extra entrances can be used as additional gas inlets (gas entry-2, Fig. 1). When performing reactive sputtering, some differences could be expected between the use of gas entry – 1 or – 2. When gas entry-1 is employed (i.e., injecting from the back side of the magnetrons), the probability to directly decompose the added gas in the plasma is greater than when gas entry-2 is used, since the gas from gas entry -1 must pass through the magnetron region.

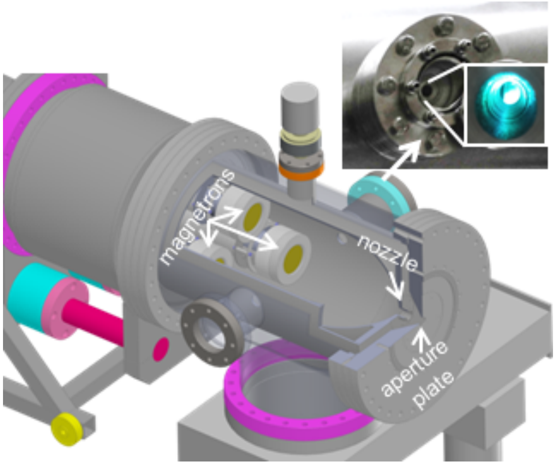

Figure S1. *Scheme of the MICS Module.*

S2. THE DIAGNOSIS MODULE

A home-made Electrostatic Quadrupole Deflector (EQD), [3] comprising a combination of Einzel lenses and an electrostatic deflector, diverts the charged particles 90° upwards when inserted into the beam path. The EQD is mounted on a XYZ translator allowing for fine alignment and retraction when not in use. It can guide the charged particles towards a quadrupole mass spectrometer QMF 200 (Oxford Applied Research Ltd.) with a mass range from 0 to 10^6 amu and a resolution of $\Delta M/M \sim 10$.

The quartz crystal microbalance (2) is mounted on a z-translator perpendicular to the beam allowing the monitoring of NP rates produced in the MICS module at different positions of the beam. The sample entry point is positioned just in front of the quartz crystal microbalance.

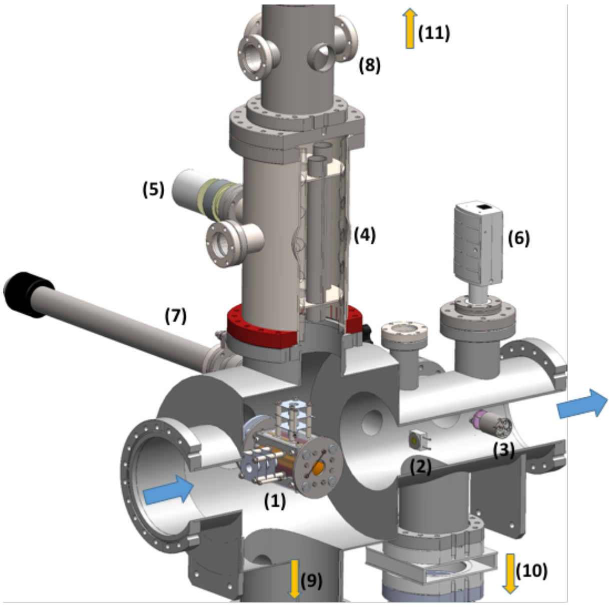

Figure S2: *The blue arrows indicate the nanoparticles advancing direction. The yellow arrows show the gas flow towards the pumps. (1) Electrostatic Quadrupole Deflector; (2) Quartz Crystal microbalance; (3) Mass spectrometer 0-200 amu (Pfeiffer); (4) Quadrupole QMF200 0 - 10^6 amu (Oxford Applied Research); (5) Pirani-Penning pressure gauge; (6) Pirani with Bayard-Alpert pressure gauges; (7) Sample entry; (8) Analysis chamber for ions and/or charged nanoparticles; (9), (10) and (11) Turbomolecular pumps.*

S3. THE OVEN MODULE

The Oven module comprises three infrared lamps (Hereaus Nano XP 2120) that emit a quasi-black body spectrum with a maximum filament temperature of ~1420 K and around 2 kW of heating power per lamp. The complete module is water cooled using a double wall construction. In addition, a water-cooled optically-thick baffle is installed between the chamber and its turbo pump to protect the pump from the IR radiation (see Figure S3a). Due to its particular configuration and the large heat load on the walls during operation, typical pressures in the Oven module cannot be measured. Diaphragms are installed both at the entrance and exit of the Oven module (see Figure S3a), which act as IR mirrors that help to maintain a uniform heat distribution in the chamber and reduces the heat load in the neighboring modules. These consist of blank copper gaskets with a central aperture of 10 mm and 12 mm at the entrance and exit ports, respectively. Moreover, these diaphragms are an essential part of the differential pumping configuration in *Stardust*. When the MICS module is operating, they prevent the gases needed for NP formation to enter the subsequent modules.

A calibration of the oven temperature was performed by measuring the near IR emission spectra of the lamps in the oven chamber. The spectra were fitted to the black body thermal emission. Figure S3b depicts the estimated temperature at different applied lamp powers using one lamp. Additionally, we checked that using three lamps the applied power and spectral radiance was increased by a factor of 3 whereas no effect was observed on the temperature.

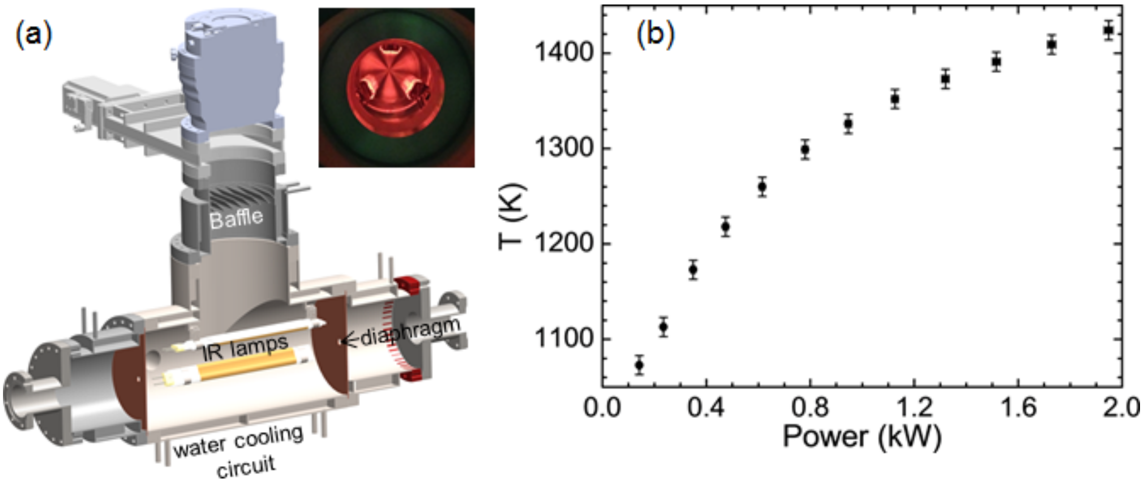

Figure S3. *(a) Cross section of schematic representation of the Oven module. The IR-lamps, baffle and diaphragms are indicated. Inset: photo of the three IR lamps turned on. (b) Temperature versus applied lamp power for one working lamp.*

S4. THE ACCELERATION MODULE

The intrinsically charged particles (for metals most of the generated NPs are negatively charged) can be filtered using deflection plates, diverting the charged nanoparticles horizontally out of the beam. To ionize the remaining neutral NPs, we use a home-built axial grid electron source, providing incident electron energies ranging from 20 eV to 300 eV. In this range of electron energies, ionization is mainly due to electron knock-off, leading to a beam of positively charged nanoparticles. The charged NPs can be accelerated using a pulsed extraction and, afterwards, can pass through an Einzel lens and steering optics. Acceleration voltages for pulsed extraction can be up to 6 kV (ISEG nhs voltage source), with

both polarities. High voltage pulsing is performed with a Behlke GHTS 60 high voltage switch and pulses are timed by a SRS DG535 Digital Delay Generator.

In order to investigate the influence of charge on the reactivity of accelerated nanoparticles, further neutralization of positively charged NPs can be performed with a low energy electron source (0 – 10 eV and 0 – 500 eV, SPECS Flood Gun FG 15/40). A second set of electrostatic deflection plates are installed after the electron source so as to remove any remaining charged NPs from the beam if required or to quantify the fraction of NPs that have been ionized.

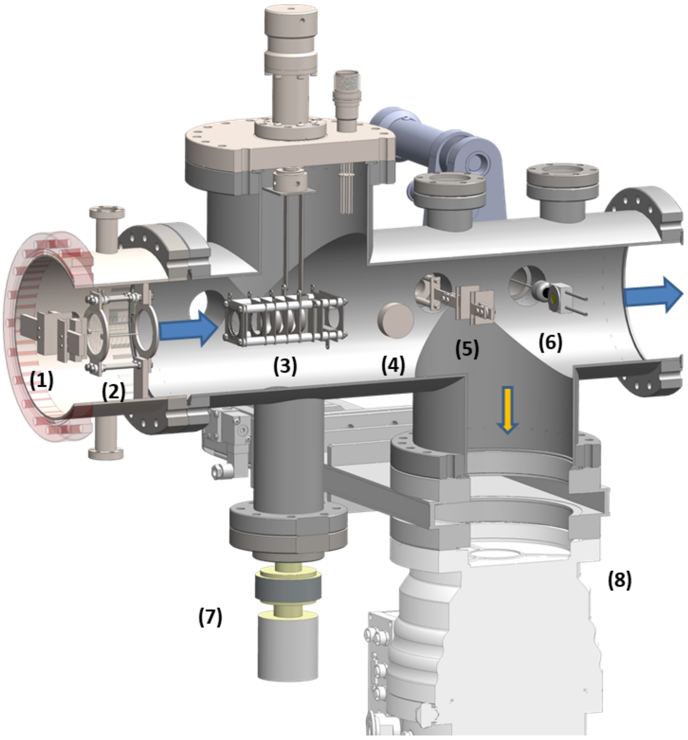

Figure S4.1. *Schematic view of the acceleration module. The blue arrows show the nanoparticles advancing direction. The yellow arrows show the gas flow towards the pumps. The numbers refer to the following instrumentation: (1) Horizontal deflection plates. (2) Axial Ionizer. (3) Ion optics module, mounted on Z-translator, consisting of acceleration plates, Einzel lens and deflection plates (horizontal plus vertical). (4) Flood gun (Oxford Applied Research Ltd.). (5) Horizontal deflection plates. (6) QCM and Faraday cup, both retractable. (7) PBR pressure gauge. (8) Turbomolecular pump.*

The Ionizer

In order to determine the efficiency of the ionizer, the charged particles were deflected before entering the ionizer, and using QCM-2 ((6) in Figure S4.1.) the rate of neutral particles was monitored using QCM-2 ((6) in Figure S4.1.). Subsequently, the neutral beam was ionized (V_e = 104 V; I_e = 5 mA), the ionized particles deflected in the next set of deflectors, and the remaining mass rate was determined. The results presented in Figure S4.2. indicate that for Cu NPs (P = 27 W; ϕ_T = 150 sccm; ϕ_{Ar} = 10 sccm; aggregation length 374 mm) the ionizer can ionize over 90 % of the mass of nanoparticles present in the beam. As most mass will be present in the largest particles, which have also the largest cross section for electron impact, one cannot extrapolate this result directly to the relative number of NPs ionized.

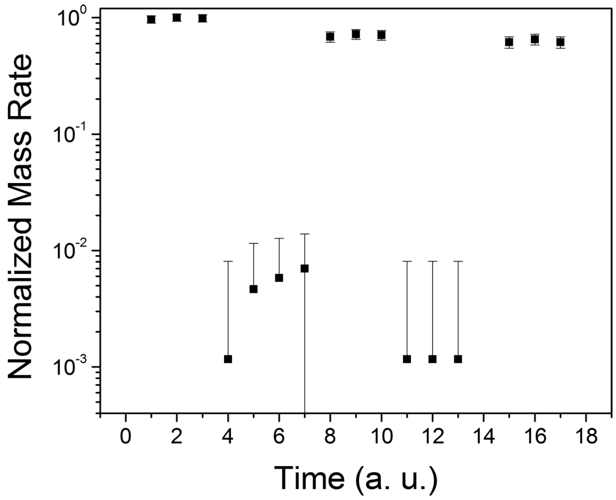

Figure S4.2. *Normalized mass rate measured by QCM-2 while turning on and off the filament of the ionizer. All other parameters were kept constant during the experiment.*

Speed calculations

Applied trigger voltage on the first set of deflection plates ((1) in Fig. S4.1): square pulse of 1 kV amplitude, 2 ms duration. Time delay was measured on the Faraday cup ((6) in Fig. S4.1)

The observed response on the Faraday cup is convoluted with the peak noise introduced by the high voltage applied on the deflection plates. The delay time was analyzed by differentiating the Faraday cup response and using a low pass filter to eliminate the high frequency noise. An average speed of 140 ± 20 m/s was found for NPs of 13 nm diameter.

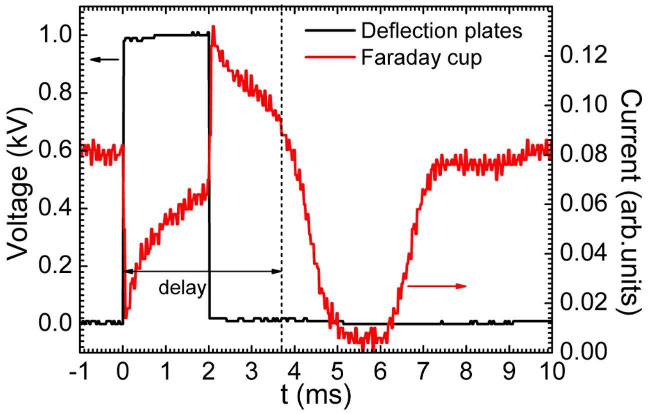

Figure S4.3. *Time evolution of the trigger voltage on the deflection plates (black) and simultaneous recording of the Faraday cup current (red). The distance between the deflection plates and the Faraday cup is 51 cm.*

S5. GAS ENTRY POINTS, GAS MIXING AND INLET SYSTEM

Stardust includes five gas entry points (depicted in Fig. 1), three of which are located in the MICS module. Gas entry-1 is located behind the magnetrons (each magnetron has two entrances here apart from the sputtering gas injection). Another gas entrance (not depicted in Fig. 1) is mainly devoted to He injection between the three magnetrons of the MICS, while the third gas entry point of the MICS module (gas entry-2) is placed halfway down the aggregation zone. Gases can also be

injected during the flight of the NPs in the Acceleration module (gas entry-3), or once deposited on a substrate in the ANA chamber (gas entry-4).

A gas mixing system has been designed to introduce gases into each module of the Stardust machine so as to promote NP-gas interactions. To this end, four identical highly controlled gas mixing systems are available along Stardust with accurate in-line monitoring of the mixture composition. Each system is comprised of three main parts as depicted in Figure S5.

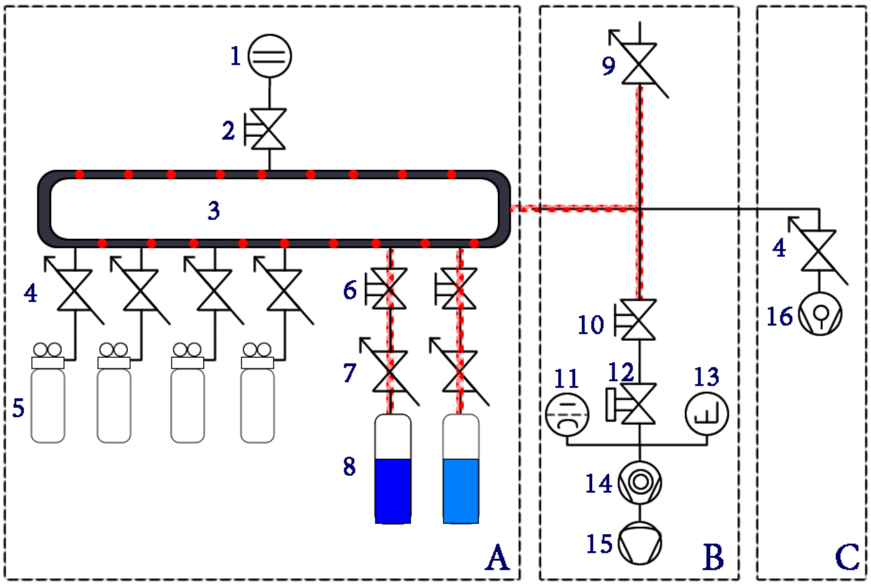

Figure S5. *Technical scheme of the gas mixing and inlet system. (a) Gas mixing chamber. (b) Gas monitoring and gas inlet connection. (c) Pumping system. 1.- Capacitive vacuum gauge; 2.- Membrane valve; 3.- Mixing chamber; 4.- Gas dosing valves; 5.- Gas bottles; 6.- Membrane valves; 7.- Needle valves; 8.- Liquid reservoirs; 9.- Leak valve; 10.- Membrane valve; 11.- Pirani-Penning vacuum gauge; 12.- Gas regulating valve; 13.- Mass spectrometer; 14.- Turbomolecular pump; 15.- Membrane vacuum pump; 16.- Rotary pump. The red dots and red lines denote the heating elements.*

The first part of the mixing system is the gas mixing chamber (3), where gas mixtures are prepared. It consists of a 3.3 l stainless steel cylinder with four gas and two liquid vapor entrances. Whilst the gas entrances are connected to gas bottles through manual gas dosing valves *EVN 116* (Pfeiffer), each liquid vapor entrance is connected to a 0.5 l stainless steel liquid reservoir through a membrane and a needle valve. Both the cylindrical chamber and the connection pipes between the liquid reservoirs and the chamber are surrounded by a 120 W heating tape and covered by insulating material in order to avoid liquid condensation on the internal walls. With this heating power, a stable and homogeneous working temperature of 90°C is achieved. In addition, a capacitive vacuum gauge is connected to the mixing chamber providing a gas-independent pressure measurement.

The next part of the system consists in the gas monitoring and gas inlet connection to the *Stardust* modules (b). To guarantee accurate control of the gas mixture composition, a quadrupole mass spectrometer *PrismaPlus QMG 220 M1* (Pfeiffer) covering a mass range of 1-100 amu is used in-line with the mixing chamber. This allows not only monitoring the gas mixture but also fine tuning of the ratio of the components in the mixture with excellent accuracy. The mass spectrometer is installed in a UHV chamber with a *HiCube 80* (Pfeiffer) pumping station, reaching a base pressure of 10^{-9} mbar. Since the gas pressure in the mixing chamber during operation is several orders of magnitude higher

than the maximum operating pressure of the mass spectrometer detectors, a regulating valve *RME 005 A* (Pfeiffer) is installed between the mixing chamber and the mass spectrometer UHV chamber. This valve allows control of the gas flow to the UHV chamber through a PID controller using the input of a Pirani-Penning gauge. Once the gas mixture is prepared, the gas flow towards the *Stardust* machine is controlled by a leak valve.

Finally, the pumping system (c) is comprised of a *DUO 3* rotary pump (Pfeiffer) and a manual gas dosing valve *EVN 116* (Pfeiffer). With this pumping system, the base pressure on the gas mixing chamber is 10^{-3} mbar. The valve acts as a throttle valve to adjust the system conductance and allows a continuous dynamic mixing of the gases. In this manner, a proper mixing of the gases is achieved in the gas mixing chamber in an overall pressure range from 10^{-2} mbar to 110 mbar (the measuring range of the capacitive vacuum gauge).

S6. PUMPING SOLUTIONS

The production of nanoparticles in the MICS module and its extraction towards *Stardust* require a powerful pumping system. The path of the gas flow from the gas inlets in the MICS to the vacuum system is made through a nozzle that communicates with the rest of the chambers (see Fig. 1). Because of this, the MICS module has optimized pumping up to 10^{-2} mbar pressure of a 35 m^3/h (Edwards XDS35i) scroll dry primary pump. With this high capacity of pumping at low pressures, the expansion necessary to drag the nanoparticles towards the exit of the MICS is favored. Attached to the MICS is a turbo molecular pump (TMP) of 1200 l/s (Pfeiffer HiPace 1200), mounted on a DN200CF flange which maximizes pumping conductivity. This combination of pumps together with a guillotine flow control valve is the necessary instrument to control the flow of particles at the exit of the MICS module. To obtain a precise pressure determination, we employ a full-range Pirani-Bayard-Alpert gauge (Pfeiffer PBR 260). Depending on the experiments performed, we have a capacitive gauge (Pfeiffer CMR 261) or Pirani-Penning full-range gauge (Pfeiffer PKR 261) connected to the lateral apertures in the aggregation zone, which gives us important information on the pressure in this chamber.

The diagnosis chamber has three strategically located Turbo pumps (TMP) (see Fig. 1). The first, with a capacity of 800l/s (Agilent Navigator 1001) is placed just at the exit of the MICS, with the second of 400 l/s placed vertically favoring the arrival of nanoparticles to the quadrupole. The third TMP located at the exit of the diagnosis chamber has a capacity of 300l/s (Pfeiffer HiPace 300). Vacuum measurements are performed through a full-range Pirani-Penning gauge (Pfeiffer PBR 260). Roughing pump is 30 m^3/h (Agilent) for the 800 l/s TMP and the 300 l/s TMPs share a 15 m^3/h Scroll (Edwards)

The Oven module uses a 700 l/s TMP (Pfeiffer HiPace 700M) coupled with a pneumatic CF-160 valve, backed by a rough pump type scroll 15 m^3/h (Edwards nXDS15i). The diaphragms installed at the entrance and exit of this module play an important role in the differential pumping of the system, as they prevent a high gas load to pass through the subsequent modules. The rough pump of the Oven Chamber is shared with the Acceleration module.

The Acceleration module is pumped by a 700 l/s Turbo pump (Pfeiffer HiPace700M) supported by a rough pump type scroll 15 m^3/h (Edwards nXDS15i). Pressures are measured by a Pirani- Bayard-Alpert full-range gauge (Pfeiffer PBR 260).

These UHV conditions are achieved by means of the combination of several vacuum pumps that can maintain in the ANA module pressures close to 10^{-11} mbar. To achieve this, ANA is pumped by a TMP of 500 l/s (Pfeiffer TMU 521), backed by a HiCube eco 80 (Pfeiffer). In addition, to improve the pump speed, ANA has a 600 l/s ion pump (Gamma Vacuum TiTan 600TV) in the same pumping channel along with a titanium sublimation pump inside. A gauge Bayard-Alpert (Lewac PM-AIG17G) measures the pressure in ANA module. For the load/lock and the differential pumping of the UPS lamp, the combination of a primary scroll pump of 6 m^3/h (Edwards nXDS6i), followed by an 80 l/s turbo (Pfeiffer HiPace 80) is used.

All modules are separated with valves, and the valve between ANA and the rest of Stardust is only opened when depositing NPs in the ANA module.

S7. ROUGHNESS ANALYSIS OF THE SUBSTRATE.

In order to obtain a detailed particle size distribution histogram, the surface roughness must be taken into account, as typical grain size from the substrate can be misinterpreted as smaller particle size. In the case of Figs. 2b and 4b and 4c, small features can be seen in the images and *a priori* could be assigned to a smaller distribution in the log-normal plots of figure 4d and 4e. Figure S6 shows a line profile on three different AFM images of surfaces of SiOx. In the image at the left-hand side the line has been traced between the grown Cu nanoparticles, just over the smallest apparent grains that can be seen. The central and right-hand side images show the same SiOx substrate without nanoparticles. It is clear that all of the line profiles obtained are on the same range of vertical distances. Typical roughness of the as received SiOx surface is in the order of 0.26±0.06nm.

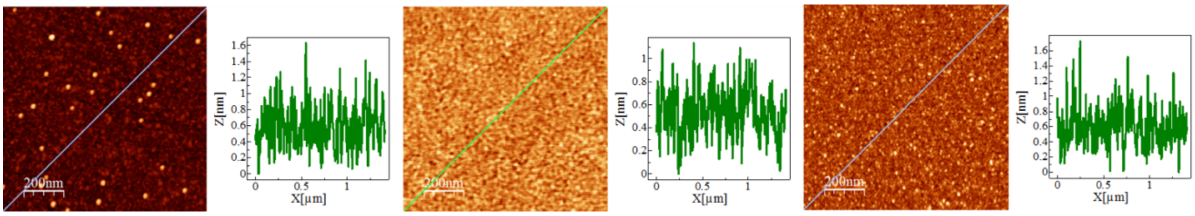

Figure S6. *AFM images and surface profile along the line on a SiOx substrate terrace. Left side: for nanoparticle covered sample, center and right side: for a clean surfaces. The surface roughness is very similar in all cases, indicating that the smallest grains observed in Figure S6, and correspondingly in Figures 2b, 4b and 4c, arise from the substrate.*
